# Infliximab can reduce mortality from 35 to 14% in critically ill patients with COVID-19: perhaps some potential confounders to consider

**DOI:** 10.1186/s13054-020-03294-7

**Published:** 2020-10-09

**Authors:** Patrick M. Honore, Leonel Barreto Gutierrez, Luc Kugener, Sebastien Redant, Rachid Attou, Andrea Gallerani, David De Bels

**Affiliations:** ICU Department, Centre Hospitalier Universitaire Brugmann-Brugmann University Hospital, Place Van Gehuchtenplein, 4, 1020 Brussels, Belgium

We read with great interest the recent article by Stallmach et al. who reported data from a small retrospective case series of patients with COVID-19 that received infliximab compared to a contemporaneous cohort that received supportive care alone [1]. Infliximab (anti-TNF) was used to target proinflammatory cytokines that are associated with deterioration of organ function and poor outcomes in patients with COVID-19 [1, 2]. The authors reported that mortality was reduced from 35% in the control group to 14% in the infliximab group [1]. We would like to make some comments. Looking at the baseline characteristics of the patients, we can see that ferritin levels in the infliximab group were almost double those in the control group (2777.4 vs 1453 μg/L) [1]. It is possible that the patients in the treated group may have had secondary hemophagocytic lymphohistiocytosis (sHLH) syndrome induced by SARS-CoV-2 [3]. The HScore, one of the tools to diagnose HLH, assigns no points to a ferritin below 2000 μg/L, while a ferritin between 2000 and 6000 μg/L adds 35 points, showing that the limit of 2000 μg/L is really crucial [3]. Due to the massive cytokine release seen in patients with the condition, HLH is considered to be a cytokine storm syndrome [3]. In potential sHLH, early use of high-dose steroids alone can be successful, consistent with the recent findings regarding the efficacy of dexamethasone in patients with severe COVID-19 receiving any form of respiratory support [4]. Other drugs such as infliximab may be life-saving in this group of patients. In conclusion, on the basis of the difference in ferritin levels between the two groups and the potential diagnosis of sHLH, the two groups in this study are not well matched [3]. A diagnosis of sHLH could explain the enormous cytokine storm responding so well to infliximab [1] and may also explain why dexamethasone is so effective in patients with severe COVID-19 [4]. The sHLH-cytokine storm may be the main reason for the rapid deterioration seen in patients with COVID-19 [3]. It is advisable to measure baseline ferritin before starting any immunological treatment in order to improve the efficacy [5].

## Data Availability

Not applicable.

## Abbreviations

TNF: Tumor necrosis factor
IL-6: Interleukin-6
sHLH: Secondary hemophagocytic lymphohistiocytosis
HS: Hematophagocytic syndrome score
