# Supplementary material for: Infliximab can reduce mortality from 35 to 14% in critically ill patients with COVID-19: perhaps some potential confounders to consider
Source: Crit Care. 2020 Oct 9;24:603. doi: 10.1186/s13054-020-03294-7 (PMC7545152; doi:10.1186/s13054-020-03294-7)
Supplement: Related texts

# Reply: Authors’ response

**Authors:** Andreas Stallmach, Andreas Kortgen, Falk Gonnert, Sina M. Coldewey, Philipp Reuken, Michael Bauer

**Affiliations:** 2 grid.275559.9 0000 0000 8517 6224 Department of Internal Medicine IV (Gastroenterology, Hepatology, and Infectious Diseases), Jena University Hospital, Jena, Germany; 3 grid.275559.9 0000 0000 8517 6224 Department of Anesthesiology and Intensive Care Medicine, Jena University Hospital, Jena, Germany; 4 grid.492124.8 0000 0001 0214 7565 Department of Anesthesiology and Intensive Care Medicine, SRH Wald-Klinikum Gera, Gera, Germany; 5 grid.275559.9 0000 0000 8517 6224 Septomics Research Center, Jena University Hospital, Jena, Germany; 6 grid.275559.9 0000 0000 8517 6224 Center for Sepsis Control and Care, Jena University Hospital, Jena, Germany

To the editor:

We thank Dr. Honore and colleagues for their thoughtful comments pointing towards a potential secondary hemophagocytic lymphohistocytosis (sHLH) characterized by pancytopenia, hyper-coagulation, acute kidney injury, and hepatobiliary dysfunction associated with COVID-19. Based on their suggestion, we applied the hemophagocytosis score (HScore) and obtained a median Hscore of 171 (136;186) for IFX-treated patients and 136 (77; 182) for controls (*p* = 0.393) reflecting that some but not all of the patients fulfilled the HScore criterium of sHLH, i.e., a score above 169. Nevertheless, all of the patients displayed a profound inflammatory state in particular in the IFX-treated patients, reflecting the experimental therapeutic approach. A recent proposal by a panel of experts suggested to screen all patients with severe COVID-19 both for a hyperinflammatory state using laboratory tests (e.g., ferritin, decreased platelet count or erythrocyte sedimentation rate) and for the HScore to identify the subgroup of patients for whom immunosuppressive therapies (e.g., infliximab or anakinra [6]) could improve outcome [7]. Whether severe COVID-19 indeed reflects a form of sHLH is still a matter of controversy; however, the evidence to support antiinflammatory interventions in the most severe cases is meanwhile supported by prospective randomized evidence for dexamethasone [4]. Though, research addressing the optimum immune modulating drug, e.g., depending on the severity of the cytokine response, is warranted [8].
